# Hospital and Patient Characteristics Associated with Neonatal Blood Stream Infection in Inpatient Care: Insights from the 2019 HCUP KID Database

**DOI:** 10.3390/children11080923

**Published:** 2024-07-30

**Authors:** Michael Samawi, Gulzar H. Shah, Linda Kimsey, Kristie C. Waterfield, Susan Hendrix

**Affiliations:** 1 Department of Health Policy and Community Health, Jiann-Ping Hsu College of Public Health, Georgia Southern University, P.O. Box 8015, Statesboro, GA 30458, USA; gshah@georgiasouthern.edu (G.H.S.); lkimsey@georgiasouthern.edu (L.K.); kwaterfield@georgiasouthern.edu (K.C.W.); 2 School of Nursing, Waters College of Health Professions, Georgia Southern University, P.O. Box 4158, Savannah, GA 31419, USA; susanhendrix@georgiasouthern.edu

**Keywords:** neonatal blood stream infection, quality improvement, pediatric care, pediatric adverse events, Kid’s Inpatient Database

## Abstract

Background: This study explores the associations between pediatric adverse events (PAEs) and both hospital and patient characteristics within the inpatient hospital setting, specifically focusing on Neonatal Blood Stream Infection (NBSI) as defined by pediatric quality indicators (PDIs) from the Agency for Healthcare Research and Quality (AHRQ). This research aims to answer questions regarding the relationship between hospital characteristics and patient demographics with the occurrence of NBSI. Methods: This study utilized discharge data from the Healthcare Cost and Utilization Project (HCUP) Kids’ Inpatient Databases (KID) for the year 2019. Bivariate and multivariate logistic regression models were employed to analyze patient-level encounters of NBSIs. The analysis examined various factors including hospital size, location, and teaching status, as well as patient-specific variables such as gender, age, race, service lines, payment sources, and major operating room procedures. Results: The results indicate that Public and Private not-for-profit hospitals showed significantly lower odds of experiencing NBSIs when compared to Private investor-owned hospitals, as did smaller, rural, and nonteaching hospitals when compared to large hospitals. Additionally, individual factors such as gender, age, race, service lines, payment sources, and types of major operating room procedures were found to have varying levels of significance in relation to NBSI. Conclusions: This study provides important insights into PAEs within the inpatient hospital setting, particularly focusing on NBSIs within the PDI framework. The findings highlight critical areas for the development of evidence-based interventions and guidelines, which are essential for clinicians and policymakers. Ultimately, this study contributes to the understanding and improvement of pediatric patient safety by emphasizing the necessity for targeted strategies to mitigate the risk of NBSI.

## 1. Introduction

Adverse events (AEs) within healthcare systems, highlighted since the seminal publication of “To Err is Human” by the Institute of Medicine in 1999 [1], continue to be a focal point despite advancements in patient safety and quality [2]. This study addresses the necessity to comprehend pediatric adverse events (PAEs) within the broader patient safety context, utilizing a pediatric quality indicator (PDI) framework for assessment. Developed as part of the Agency for Healthcare Research and Quality’s (AHRQ) efforts to standardize healthcare quality measurement, PDIs specifically target preventable complications and iatrogenic events in pediatric patients, as well as preventable hospitalizations for children [3]. Originally introduced in 2006 to distinguish them from patient quality indicators (PQIs), PDIs have evolved with refinements including risk adjustments, reference populations, and ICD-10 coding [3]. These indicators operate at both area and hospital levels, with hospital-level PDIs capturing potentially preventable complications or adverse events following medical conditions or procedures. This study relies on the classification provided by the AHRQ to investigate, measure, and mitigate NBSIs, emphasizing the utilization of hospital-level PDIs for comprehensive understanding and improvement measures.

The incidence of AEs, often conflated with medical errors, has been a subject of debate and scrutiny [4,5,6]. Studies have estimated AEs to be a leading cause of death in the United States, with systemic issues contributing significantly to their occurrence [4,6]. While the focus has shifted towards understanding and mitigating systemic flaws rather than blaming individuals, challenges persist in identifying AEs and assessing their preventability, particularly within pediatric healthcare settings [6].

In pediatric healthcare, AEs pose significant challenges, with studies reporting varying rates depending on patient populations and settings [7,8,9]. PAEs have been linked to substantial morbidity and mortality, with certain patient groups at higher risk [7]. Despite efforts to address these issues, the rates of PAEs have not decreased over time, necessitating ongoing efforts to improve patient safety through comprehensive strategies [10].

Neonatal Blood Stream Infections (NBSIs) are a critical area of concern within pediatric healthcare settings. The AHRQ defines and measures NBSIs under the PDI NQI 03, which for the year 2019 had an incidence rate of 20.23 per 1000 discharges observed from the total population of the State Inpatient Database [3]. Despite advances in medical care, NBSIs remain a significant cause of morbidity and mortality among neonates, with various risk factors including low birth weight, prolonged rupture of membranes, and mechanical ventilation [11]. Studies have identified common pathogens such as Group B Streptococcus and Escherichia coli as contributing to NBSIs, with preterm infants being particularly vulnerable [12]. Additionally, the rise of multidrug-resistant Gram-negative infections underscores the urgency in implementing both infection control measures and judicious antibiotic use [13]. Research has explored the effectiveness of central line “bundles” in reducing different types of NBSIs in neonatal intensive care units, highlighting the importance of evidence-based interventions in improving patient outcomes [14]. By focusing on NQI 03, the PDI that measures NBSIs, this study aims to provide data capable of informing evidence-based recommendations for quality improvement efforts in pediatric inpatient settings.

This study utilizes the 2019 HCUP KID database to assess one PAE, NBSIs, recognizing the importance of current and comprehensive data in understanding healthcare trends. The study also relies on the AHRQ hospital-level indicator NQI 03, measured at the patient encounter level to gauge patient harm and safety. By focusing on a specific PDI and utilizing the most recently available nationwide database, this study aims to overcome disparities in AE reporting, particularly in pediatric populations, underscoring the need for comprehensive research and interventions.

The study aims to address this knowledge gap by utilizing a high-incidence PDI as an overview of overall quality and pediatric patient safety. Specifically, it addresses the following research questions:

1. Are specific hospital characteristics (region, teaching status, rurality, ownership, size) associated with NQI 03?
2. Are specific patient characteristics (age, gender, payor, race, previous major operation, service line of provider associated with hospital visits) associated with NQI 03?

This study is purely observational and descriptive. It does not involve any intervention or manipulation of variables. Rather, it aims to comprehensively investigate PAEs, specifically NBSIs, within the framework of PDIs developed by the AHRQ. The objective is to utilize existing data to understand the incidence and associated factors of NBSIs in pediatric inpatient settings, ultimately informing evidence-based recommendations for quality improvement efforts.

## 2. Materials and Methods

This observational study employed a population-based retrospective cohort design. We extracted data from the HCUP KID dataset, 2019, obtained from the AHRQ [15]. HCUP data are created for billing and research purposes and are based on administrative data to inform policies nationwide [15]. The KID is the largest public pediatric inpatient, all-payer database that contains approximately 3 million unweighted pediatric discharges each year [15].

This study’s design allows for the measurement of outcomes and the relationship between variables. The population set available for the year 2019 encompasses national estimates of hospital inpatient stays for patients 21 and younger [15]. The number of states included in the KID database is 48, plus the District of Columbia, sampled from 4000 U.S. community hospitals including non-federal, short-term, specialist, and general hospitals but excluding rehabilitation hospitals and those as attachment units of other institutions [15]. The KID database has been available every three years beginning from 1997 to 2012 and from 2016 to 2019; however, it was not available for the year 2015 due to the transition to ICD-10-CM-PCS coding, which was utilized for this study [15]. The KID data file is structured around two elements: the discharge-level files, which include core files, severity files, and diagnosis and procedure group files, and the hospital-level files, which include information on hospital characteristics [15].

### 2.1. Variables

The main dependent variable in this study is whether Neonatal Blood Stream Infection occurred (Yes = 1; No = 0). The independent variables for this study were divided into two main categories, hospital characteristics and patient characteristics, as follows:

- Hospital characteristics: hospital bed size (coded as 1: Small, 2: Medium, or 3: Large); hospital location (1: Rural, 2: Urban nonteaching, or 3: Urban teaching); hospital region (1: Northeast, 2: Midwest, 3: South, or 4: West); and hospital ownership (1: Government, non-federal (Public), 2: Private, not-for-profit (voluntary), or 3: Private, investor-owned (proprietary)).
- Patient characteristics included gender (0: Male, 1: Female); race (1: White, 2: Black, 3: Hispanic, 4: Asian/Pacific Islander, 5: Native American, 6: Other); service line (1: Maternal and Neonatal, 2: Mental health/Substance use, 3: Injury, 4: Surgical, 5: Medical); payment type (1: Medicare, 2: Medicaid, 3: Private Insurance, 4: Self-pay, 5: No charge, 6: Other); and operations on record (0: No major operating room procedure on record, or 1: Major operating room procedure on record).

The selection of independent variables in this study was deliberate and informed by existing research that has identified notable variations in the incidence of adverse events stemming from PAEs, particularly in relation to central line-associated blood stream infection (CLABSI) and unplanned extubation (UE) [16]. Prior investigations have delineated disparities across racial and ethnic demographics, with Multiracial Hispanic and Combined Hispanic and Native Hawaiian or other Pacific Islander individuals exhibiting CLABSI rates ranging from 2.6 to 3.6 standard deviations above baseline values [16]. Similarly, Black or African American patients demonstrated UE rates elevated by 3.2 to 4.4 standard deviations [16]. Conversely, White patients displayed significantly lower rates of both adverse events compared to the baseline [16]. Moreover, given emerging evidence of systematic inequities, this study also endeavors to explore potential discrepancies in PAE reporting based on variables such as hospital location, payment modality, and specific medical services rendered [17]. This multifaceted approach aims to deepen our understanding of the complex interplay between demographic factors and adverse outcomes associated with PAEs using the most comprehensive dataset available, thereby informing more targeted interventions and policy measures.

The data element PCLASS_ORPROC denotes the presence of a major operating room procedure documented on a discharge record, as determined by the Procedure Classes Refined for ICD-10-PCS [18]. These procedures are categorized into four classes: Minor Diagnostic, Minor Therapeutic, Major Diagnostic, and Major Therapeutic [18]. Major procedures are those deemed valid operating room procedures by the Diagnosis-Related Group (DRG), performed either for diagnostic or therapeutic purposes. If the discharge record included at least one major diagnostic or major therapeutic procedure, PCLASS_ORPROC was designated as 1 [18]. Prior to the 2020 data year, the major operating room procedure indicator was stored in the data element I10_ORPROC. Further details on Procedure Class can be found under Tools & Software on the HCUP-US website [18].

The classification of discharges into distinct hospitalization types, or service lines, follows a hierarchical order encompassing five categories: Maternal/Neonatal, Mental health/Substance abuse, Injury, Surgical, and Medical [18]. The criteria for delineating these hospitalization types are subject to variation across different data years. Specifically, starting from the data year 2019, the definitions for each hospitalization type are as follows:

(1) Maternal and neonatal discharges are identified through specific Major Diagnostic Categories (MDCs), namely MDC 14 (Pregnancy, Childbirth, and Puerperium) and MDC 15 (Newborn and Other Neonates—Perinatal Period) [18].
(2) Mental health/substance use discharges are characterized by the presence of diagnoses falling under MDC 19 (Mental Diseases and Disorders) and MDC 20 (Alcohol/Drug Use or Induced Mental Disorders) [18].
(3) Injury discharges are identified using a predefined screen, involving Clinical Classification Software Refined (CCSR) categories for the principal ICD-10-CM diagnosis, specifically ranging from INJ001 to INJ027 and INJ032 [18].
(4) Surgical discharges are determined by the presence of a surgical Diagnosis-Related Group (DRG), consistent with the definition employed prior to the data year 2019. The DRG assignment process initially places the discharge into an MDC based on the principal diagnosis, followed by the assessment of the qualifying procedure codes for operating room procedures within each MDC. If such procedures are involved, the discharge is categorized into a surgical DRG within the corresponding MDC category [18].
(5) Discharges that do not meet the criteria for the Maternal/Neonatal, Mental health/substance abuse, Injury, or Surgical categories are classified as Medical discharges. In cases where the DRG does not provide sufficient information for classification as medical or surgical, discharges are typically assumed to be Medical. It is noteworthy that discharges with a principal diagnosis of injury or poisoning by intentional self-harm are categorized under the Medical service line rather than the Mental health/Substance use or Injury service lines [18].

The hospital’s bed size category (H_BEDSZ) is defined as a nested variable within the combined classification of hospital location and teaching status (H_LOCTCH) [18]. This classification system categorizes hospitals based on their geographical location and educational function. Hospitals are stratified into three location types: rural, urban nonteaching, and urban teaching, and three teaching status categories: small, medium, and large [18]. Each combination of location and teaching status corresponds to a specific bed size range. In rural settings, hospitals are categorized based on bed capacity as small (1–49 beds), medium (50–99 beds), or large (100 or more beds) [18]. Urban nonteaching hospitals are similarly categorized with small, medium, and large bed size ranges (1–99 beds, 100–199 beds, and 200 or more beds, respectively) [18]. Urban teaching hospitals, which typically have larger capacities, are categorized into small (1–299 beds), medium (300–499 beds), and large (500 or more beds) bed size categories [18].

The selection of the hospital’s location and ownership/control categories as stratifying variables is pivotal due to observed significant regional practice pattern disparities and divergent institutional missions influenced by ownership/control structures [18]. Geographical location serves as a crucial determinant, as evidenced by substantial variations in practice patterns across regions; for instance, hospitals on the East Coast typically exhibit longer lengths of stay compared to those on the West Coast [18]. Additionally, ownership/control classification, sourced from the AHA Annual Survey of Hospitals, encompasses government non-federal (public), private not-for-profit (voluntary), and private investor-owned (proprietary) categories, each associated with distinct institutional missions and responses to governmental regulations and policies [18]. Such diversity in ownership/control status underscores the necessity of these variables in understanding and contextualizing healthcare delivery dynamics, facilitating comprehensive analyses of healthcare outcomes and resource utilization patterns across different hospital settings [18]. Appendix B further contains explorations relevant to the variables and their definitions. 

### 2.2. Analysis

The analyses were conducted using the application of the AHRQ PDIs to the HUCP KID database for the year 2019, for which the AHRQ QI SAS software that was created by the AHRQ for this purpose must be used [3]. For this study, the SAS QI^®^ v2020 was used, which was adopted to be used with SAS Version 9.4 as a personal computer-based, single-user application, along with the specific population file for the v2020 provided by AHRQ [3]. To model the dichotomous dependent variables, logistic regression analyses were used. We computed odds ratios and adjusted odds ratios. The statistical models utilized inpatient discharges as the units of analysis. Logistic regression allows for the estimation of odds ratios, which indicate the likelihood of an event occurring (e.g., the occurrence of a PDI) based on the values of the independent variables. We tested the independence by running the correlation between pairs of independent variables. The correlation matrices show the highest correlation coefficients of age with I10_SERVICELINE and age with PCLASS_ORPROC to be slightly above 0.3 while none of the pairs of variables had a correlation coefficient above 0.5 (Appendix A). This model, alongside the software, provided important insights into the associations between these variables to inform strategies for quality improvement and patient safety, as well as guide future research and interventions in pediatric healthcare settings. This study was exempted from full institutional review under protocol H23359 since it utilizes secondary data wherein human subjects cannot be identified.

## 3. Results

The descriptive analysis of the HCUP KID dataset (2019) provides valuable insights into the distribution and characteristics of the hospitals and patients in the sample. The analysis includes independent variables: hospital characteristics such as bed size, location, region, and ownership; patient characteristics like gender, race, service line, payment source, and the presence of surgical operations. Together, these findings serve as a foundation for understanding the profiles of the hospitals and patients under consideration and pave the way for further analysis and interpretation of the data.

Most hospitals in the sample were classified as large (60.7%), followed by medium (24.0%) and small (15.2%), in terms of bed size. Regarding location, most hospitals were classified as urban teaching hospitals (82.3%), followed by urban nonteaching hospitals (11.6%) and rural hospitals (6.1%). In terms of hospital region, the largest proportion was in the South (38.3%), followed by the West (22.0%), Midwest (22.6%), and Northeast (17.1%). Hospital ownership was predominantly Private, not-for-profit entities (77.1%), with Public hospitals accounting for 11.8% and Private, investor-owned hospitals accounting for 11.0%.

The mean age of patients at admission was 5.58 years, with a minimum age of 0 years and a maximum age of 20 years (standard deviation = 7.574). Approximately 51.4% of patients in the sample were female, while 48.6% were male. The largest racial group among patients was White (45.6%), followed by Black (16.6%), Hispanic (19.7%), Asian/Pacific Islander (4.0%), Native American (0.9%), and Other (6.1%). The most common service line for hospitals in the sample was Maternal and Neonatal (55.6%), followed by Medical (27.4%), Mental health/Substance use (6.8%), Surgical (7.1%), and Injury (3.2%). Regarding payment source, most patients had Medicaid (50.7%), followed by Private Insurance (41.1%), Self-pay (4.3%), Medicare (0.3%), Other (3.3%), and No Charge (0.1%). Most patients in the sample did not have any operation recorded (88.0%), while the remaining patients (12.0%) underwent a major operation.

The QI in question (NQI 03) was taken at the numerator level to produce events/occurrences rather than a rate. The analysis was conducted using the FREQ Procedure on the KID 2019 database, specifically examining the neonatal population segmented by age (AGE_NEONATE), revealing that most neonates, approximately 78.61%, did not display NBSIs, amounting to 1,833,049 cases (Table 1). Conversely, a significant proportion of the neonatal population, accounting for 21.39%, did exhibit NBSIs, totaling 498,829 cases. These statistics offer valuable insights into the occurrence of NBSIs among neonates in the specified year’s database as it solely focuses on the neonatal population and not pediatric patients. It is worth noting that there were 14 missing cases in this analysis.

Overall, these descriptive statistics provided a comprehensive overview of both hospital and patient distribution and characteristics within our sample. These findings helped us draw up a map of our understanding of hospital and patient profiles under consideration and lay the foundation for further analysis and interpretation of the data. For the specific group sizes of each variable present in the database, please refer to Appendix C. 

In the bivariate logistic regression analysis, several patient and hospital characteristics were examined for their association with events of NBSIs. With the sample population being restricted to the neonate variable, the results revealed distinct relationships that pertained to that age of classification. 

For patient characteristics (Table 2), gender did not reveal a significant association (*p* = 0.5876), indicating that there was no significant difference in the odds of infection between genders.

Regarding race, it was found to be strongly associated with NBSI events (*p* < 0.0001). The variable showed variations among different racial categories; when compared to the reference category “White”, all other races displayed significantly higher odds ratios. For instance, the OR for Black patients was 1.453 (95% CI: 1.440–1.467), indicating 45.3% higher odds of infection compared to Whites, whereas Hispanic patients (OR = 1.351; 95% CI: 1.339–1.363) showed 35.1% higher odds of infection compared to Whites. Moreover, while Asian/Pacific islanders (OR = 1.421; 95% CI: 14.01–1.442) showed 42.1% higher odds of infection compared to Whites, Native Americans (OR = 0.530; 95% CI: 0.508–0.533) showed an inverse relationship with 53% lower odds of infection compared to Whites. Other races (OR = 1.314; 95% CI: 1.297–1.331) showed 31.4% increased odds of infection.

In terms of the service line, indicating the type of medical service provided, a significant association with NBSIs was found (*p* < 0.0001, *p* < 0.5 Injury), except for Mental health/Substance abuse (*p* = 0.2912) when compared to the “Medical” service line. The odds ratio (OR) for the “Surgical” service line was particularly high at 1.783 (95% CI: 1.686–1.886), indicating a significantly higher risk of 78.3% compared to the reference category Medical. Maternal and Neonatal services showed the lowest odds of infection (OR = 0.585; 95% CI: 0.567–0.603) with 58.5% lower odds of infection when compared to Medical, followed by Injury (OR = 1.266; 95% CI: 1.058–1.515), with higher odds of infection amounting to 26.6% when compared to Medical. 

Regarding payment source, significance was associated with NBSIs when compared to “Self-pay” (*p* < 0.0001), where Medicare (OR = 0.668; 95% CI: 0.619–0.720) showed 33.2% lower odds of infection. No charge (OR = 0.579; 95% CI: 0.523–0.640) also showed a 42.1% lower odds of infection when compared to Self-pay, whereas Private Insurance (OR = 1.526; 95% CI: 1.501–1.551), Medicaid (OR = 1.305; 95% CI: 1.283–1.326), and Other (OR = 1.577; 95% CI: 1.539–1.617) all showed higher odds of infection (52.6%, 30.5%, 57.7%, respectively) when compared to the odds of infection of Self-pay patients. 

Regarding major operating room procedure, neonate patients had significantly higher odds (OR = 1.980; 95% CI: 1.932–2.029) of infection when compared to patients without such procedures. This amounted to a nearly twofold 98% higher odds of infection, drawing attention to the concentration of risk present in operative circumstances. 

For hospital characteristics (Table 3), all were found to be significantly associated with NBSIs (*p* < 0.0001). Hospital bed size demonstrated a significant association, with small-sized hospitals showing an odds ratio of 0.442 (95% CI: 0.438–0.446), indicating a 55.8% lower likelihood of infection events when compared to the reference, large-sized hospitals. Medium-sized hospitals had an OR of 0.755 (95% CI: 0.749–0.760), representing a 24.5% lower risk. These findings suggest that larger hospitals may face higher infection risks compared to smaller and medium-sized hospitals.

Hospital location also displayed a significant relationship with NBSIs. Rural hospitals had the lowest OR of 0.074 (95% CI: 0.072–0.075), indicating a 92.6% lower risk of infection events compared to urban teaching hospitals (reference category). Urban nonteaching hospitals had an OR of 0.466 (95% CI: 0.461–0.470), signifying a 53.4% lower risk, when compared to urban teaching hospitals, showcasing the importance of considering hospital location when looking at neonatal infection.

Regarding hospital location, the odds ratios for the Northeast, Midwest, and South regions were estimated to be 0.929 (95% CI: 0.919–0.939), 0.787 (95% CI: 0.779–0.795), and 1.108 (95% CI: 1.099–1.118), respectively. These figures represent a 7.1% lower risk in the Northeast, a 21.3% lower risk in the Midwest, and a 10.8% higher risk in the South compared to the West (reference category).

Furthermore, hospital ownership exhibited a significant relationship with NBSIs. Public hospitals had an odds ratio of 0.725 (95% CI: 0.715–0.735), indicating a 27.5% lower risk compared to Private investor-owned hospitals. Private, not-for-profit hospitals had an odds ratio of 1.025 (95% CI: 1.015–1.035), suggesting a 2.5% increased risk when compared to Private investor-owned hospitals. These results imply that hospital ownership may influence infection risks, with public hospitals showing a lower risk compared to Private, not-for-profit hospitals.

Following along with the multivariate analysis (Table 4), this study sought to present associations with the variables while controlling for other factors. The associations for patient characteristics remained largely consistent, though gender (female) did show a significant association (*p* = 0.0158) with an adjusted odds ratio (AOR) of 1.009 (95% CI: 1.002–1.015), suggesting a 0.9% increased risk compared to males. This showed that there is a statistically significant difference between males and females in terms of infection odds when other variables are controlled for. 

In terms of race, all races showed significant associations with NBSIs when compared to White race and controlling for other variables. Being Black was associated with 16.6% higher odds of infection (AOR = 1.166; 95% CI: 1.154–1.178) when compared to Whites and controlling for other variables, while Hispanics had 7.7% higher odds than Whites when controlling for other variables (AOR = 1.077; 95% CI: 1.066–1.087) and Asian/Pacific Islanders had 4.3% higher odds (AOR = 1.043; 95% CI: 0.962–0.990) when compared to White patients and controlling for other variables. The category “Others” was associated with 5.2% higher odds when compared to Whites (AOR = 1.052; 95% CI: 1.038–1.066), while Native Americans (AOR = 0.795; 95% CI: 0.761–0.831) showed an inverse relationship with 20.5% lower odds of infection when compared to Whites and controlling for other variables, remaining consistent with the findings of the bivariate analysis. These findings highlight the prominence of certain racial disparities in the risk of infection. 

Examining the service line, Mental health/Substance use (*p* = 0.5706) remained insignificant as in the bivariate analysis; however, the “Injury” service line (*p* = 0.6389) also became insignificant when compared to the “Medical” service line and controlling for other variables. Maternal and Neonatal services, however, showed a significant association with NBSI events with an AOR of 0.682; 95% CI: 0.661–0.704, indicating 31.8% lower odds of infection when compared to the Medical service line and controlling for other variables. The Surgical service line also showed significance, with an AOR of 0.929; 95% CI: 0.871–0.991 and 7.1% lower odds of infection than the Medical service line and controlling for other variables. 

Moving on to payment sources, when compared to Self-pay and controlling for other variables, all payment sources showed significance (*p* < 0.0001) with NBSI events. Both Medicare (AOR = 0.672; 95% CI: 0.621–0.727) and No charge (AOR = 0.670; 95% CI: 0.603–0.744) displayed 32.8% lower odds of infection. On the other hand, Medicaid (AOR = 1.079; 95% CI: 1.060–1.098), Private Insurance (AOR = 1.228; 95% CI: 1.207–1.250), and Other (AOR = 1.361; 95% CI: 1.326–1.397) showed higher odds of infection (7.9%, 22.8%, 36.1%, respectively). These findings mirror those of the bivariate analysis and suggest that hospitals accepting certain payment sources may confer a protective effect against infection.

Meanwhile, a major operation on record (AOR = 1.457; 95% CI: 1.414–1.501) was associated with 45.7% higher odds of infection when compared to no major operation on record and controlling for other variables, further mirroring bivariate findings, and consistently showing the vulnerability of repeat patients to infection. 

Among hospital characteristics, small and medium bed-sized hospitals (AOR = 0.421; 95% CI: 0.417–0.426, AOR = 0.730 95% CI: 0.724–0.736, respectively) consistently showed significantly (*p* < 0.0001) lower odds of infection (57.9%, 27%, respectively) when compared to large hospitals and controlling for other variables, which could be related to the type of services offered in small- and medium-sized hospitals as opposed to larger ones. Nonetheless, hospital location also played a significant role (*p* < 0.0001), with rural (AOR = 0.672; 95% CI: 0.621–0.727) and urban nonteaching hospitals (AOR = 0.672; 95% CI: 0.621–0.727) both showing lower odds (92.4%, 53.9%, respectively) of infection when compared to urban teaching hospitals. These results were consistent with the bivariate analysis and displayed an increased risk in environments associated with urbanism and teaching.

When examining hospital region (all *p* < 0.0001), the South region (AOR = 1.141; 95% CI: 1.131–1.152) was associated with 14.1% higher odds of infection compared to the reference category (West region), whereas the Northeast (AOR = 0.816; 95% CI: 0.807–0.826), and the Midwest (AOR = 0.899; 95% CI: 0.889–0.909) were found to be significantly associated with lower odds of infection (18.4%, 10.1%, respectively) when compared to the West and controlling for other variables. 

Regarding hospital ownership, both Public (AOR = 0.733; 95% CI: 0.723–0.744) and Private not-for-profit (AOR = 0.948; 95% CI: 0.948–0.968) hospitals were found to be significantly associated (*p* < 0.0001) with lower odds of infection (26.7%, 4.2%, respectively) than Private investor-owned hospitals when controlling for other variables. It is of note that the relationship associated with Private not-for-profit hospitals became inverse when controlling for other variables in the multivariate analysis, suggesting that there are other factors impacting the relationship with infection events in these types of hospitals. 

The findings of the above bivariate and multivariate logistic regression analyses examined the association between various patient and hospital characteristics and the occurrence of NBSI events in neonates. In the bivariate analysis, gender did not show a significant association with NBSI events, while race, service line, payment source, major operating room procedure, hospital bed size, hospital location, hospital region, and hospital ownership were all significantly associated with NBSI events. When controlling for other factors in the multivariate analysis, the associations for patient characteristics remained largely consistent. Gender (female) showed a significant association with a slightly increased risk of NBSIs compared to males. Race, service line, payment source, major operating room procedure, hospital bed size, hospital location, hospital region, and hospital ownership all maintained significant associations with NBSI events. However, there were some changes in the magnitude of the associations in the multivariate analysis compared to the bivariate analysis.

**Figure 1:**
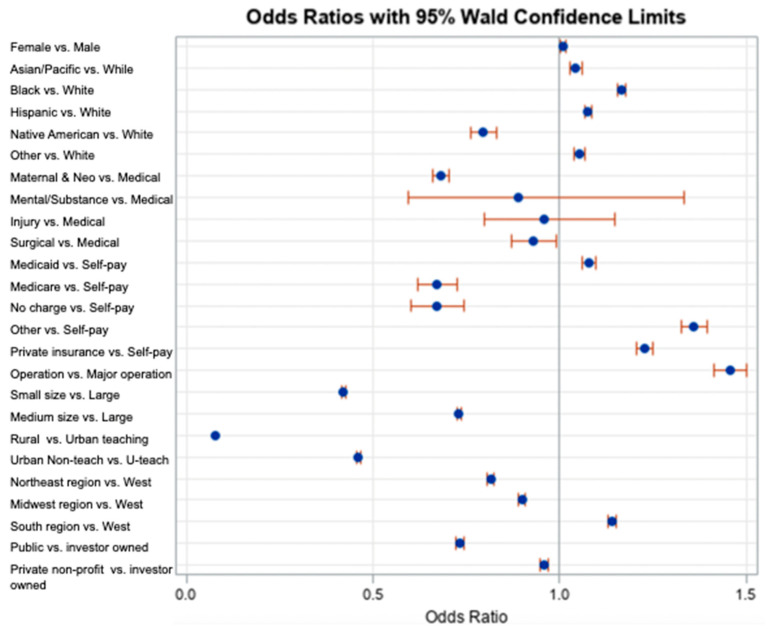
Odds ratio with 95% Wald confidence limits associated with NBSIs (multivariate). Blue dots indicate AOR and Orange lines indicate 95% Wald Confidence Limits.

Both the bivariate (Table 2 and Table 3) and multivariate (Table 4) analyses showed that various patient and hospital characteristics have significant associations with NBSI events, suggesting that these characteristics play a role in the occurrence of NBSIs and should be considered when considering preventative measures. (Figure 1) showcases the relationships between characteristics and infection risk as well as the reliability of the outcomes. Nonetheless, there were several possible reasons for the relationships observed between the variables and NBSI events.

For patient characteristics, the significant association between race and NBSI events may be due to differences in genetic susceptibility, access to healthcare, or exposure to risk factors among different racial groups. The associations with payment source may reflect differences in healthcare access, quality of care, or underlying health conditions associated with hospitals that accept different types of insurance.

In terms of hospital characteristics, the significant associations with hospital bed size and location could be attributed to differences in resources, staffing, infection control measures, or patient volume. The relationships with hospital region may reflect regional differences in infection control practices, healthcare infrastructure, or patient demographics and confirm the variances noticed in medical practices across regions [18]. The association with hospital ownership may be influenced by differences in funding, management practices, or quality control measures.

Overall, these findings suggest that patient and hospital characteristics play a significant role in NBSI events and addressing these factors could help reduce the risk of infection in neonates.

## 4. Discussion

This study focused on assessing the characteristics associated with NQI 03 (NBSIs) in pediatric patients within inpatient hospital settings. Using discharge data from the 2019 HCUP KID database, this study aimed to analyze the association of hospital and patient characteristics with NBSIs at the patient level, employing logistic regression for analysis.

This study also aimed to evaluate the validity of utilizing NQI 03 as a framework for understanding NBSIs and designing targeted interventions. The findings highlight the multifactorial nature of NBSIs and emphasize the importance of considering patient demographics and hospital characteristics when assessing the risk of NBSIs. This study contributes to the existing literature by providing empirical evidence on the relationship between these factors and the occurrence of NBSIs.

One significant finding of this study was the role of gender in predicting NBSIs. Gender exhibited some interesting significance in predicting NBSIs, considering that it was not a significant predictor in the bivariate analysis but became so in the multivariate analysis, implying that the association between gender and NBSIs is masked or confounded by the presence of other variables. This indicates that it may indeed play a role in screening, or that physiological factors such as the types of infection that female neonates are susceptible to or other biological patterns may disproportionately impact female patients when larger contexts are considered. Environmental or social factors such as differences in caregiving practices or exposure to pathogens in different settings may also play a role when considered within context. Understanding the interplay between biological, social, and environmental factors is crucial for elucidating any gender disparities in infection rates in NBSIs despite the mechanism by which gender impacts infection occurrence remains unclear. Moreover, when taking into consideration other types of PAEs that have been studied with the male gender found as associated with a higher likelihood of AEs, the findings of this study add nuance to understanding the targeted role of gender in PAEs and specifically that of neonates [19]. 

It is noteworthy that a recent study using the same database and framework as this article examined KID hospitalizations to identify disparities in safety events based on race, ethnicity, and payer status. The study found that Black and Hispanic children exhibited significant disparities in five out of seven PDIs, with the most pronounced disparity in postoperative sepsis among Black patients (AOR, 1.55 [1.38–1.73]) [20]. Similarly, Medicaid-covered patients had significantly higher odds in four out of seven PDIs, again with the largest disparity in postoperative sepsis [20]. Stratified analyses revealed persistent racial and ethnic disparities even among privately insured children [20]. This study, however, focused only on two variables (race and payer status) against the backdrop of seven PDIs. While our study also showed similar disparities among Black neonates, the association was not as extensive. Nevertheless, our study uncovered consistent disparities among racial groups, with differential odds of experiencing NBSIs. Given the large sample size of the database (Appendix C) and its status as the most comprehensive nationwide database, these findings confirm the racial disparities observed across various studies regarding PAEs and emphasize the need for further research into the underlying factors contributing to these disparities.

In contrast to the aforementioned study, our findings showed that the payment source also played a significant role. Medicaid and Medicare were associated with relatively lower or inverse risks for NBSIs. This is an intriguing finding as it suggests that the impact of the payment source depends on various factors and characteristics that, when considered together, change the understanding of infection risk in a broader context. While many studies have highlighted the impact of financial stress on hospital quality of care and patient outcomes [21], this study suggests that private insurance and other payment sources are more significantly associated with the occurrence of NBSIs. Consequently, the results challenge conventional assumptions and underscore the importance of analyzing the various payment types that hospitals predominantly rely upon for resource utilization and allocation, rather than solely focusing on the underlying socioeconomic and healthcare access disparities that may affect infection risk, particularly in the context of Medicaid expansion under the Affordable Care Act [22]. This necessitates the customization of quality measures to align with the payment types that hospitals predominantly use for resource management and allocation.

Furthermore, the type of medical service provided was significantly associated with NBSI risks, with the Maternal and Neonatal lines carrying consistently lower risks for NBSIs across the analysis, indicating a lower association with typical procedures classified as MDC 14 and MDC 15. However, of interest may be the observation that the Injury service line lost significance when controlling for other variables and the Surgical line developed an inverse relationship when controlling for other variables and comparing to Medical, indicating that surgical patients may have lower odds of experiencing NBSI events when other factors are taken into account. This finding contradicts the expectation that surgical interventions would inherently carry higher infection risks due to their invasive nature. Therefore, it suggests that factors such as stringent infection control protocols or specialized care practices in surgical settings may effectively mitigate infection risks among mothers and/or neonates undergoing surgical procedures. These findings highlight the complexity of the relationship between service lines and NBSI occurrence and underscore the importance of considering the interplay between various patient and hospital factors in understanding infection risks and informing targeted interventions and quality improvement efforts. 

Additionally, hospital characteristics such as size, teaching status, rurality, region, and ownership exhibited varying associations with NBSI risks. These findings underscore the influence of healthcare infrastructure and organizational resources on NBSI occurrence, necessitating tailored interventions based on hospital types and population trends. It is noteworthy that the results of this study align with previous research indicating that the acquisition of hospitals by private equity firms tends to correlate with heightened occurrences of hospital-acquired adverse events [23]. This trend persists even when considering the probability of a lower-risk demographic among Medicare beneficiaries admitted to such hospitals, implying a lower standard of inpatient care quality [23].

Moreover, a study by Jackson et al. (2019) documents a concerning trend where physicians often deviate from WHO guidelines when diagnosing and treating NBSIs [24]. This deviation can result in missed or misidentified cases of NBSIs, further complicating our understanding of its incidence and associated factors [24]. Our findings reinforce the necessity for healthcare providers to follow standardized diagnostic guidelines rigorously. Adhering to these guidelines is crucial to ensure accurate diagnosis and appropriate treatment, ultimately enhancing patient safety and care quality for neonates.

### 4.1. Public Health Implications and Recommendations

Building upon existing research, this study affirms the validity of using NQI 03 as a framework for understanding and addressing NBSIs in pediatric care. The findings underscore the importance of targeted interventions to enhance pediatric patient safety, with implications for addressing health disparities, improving infection control measures, considering regional variations, and investigating hospital ownership.

### 4.2. Strengths and Limitations

Our study relies on the HCUP KID’s dataset, which, while offering the strength of a large and comprehensive database enabling extensive analysis and the use of logistic regression to identify independent associations, has intrinsic limitations. Notably, the KID dataset does not include data from all states and is restricted to hospitals that provide care to patients aged 20 years or younger, potentially resulting in a higher estimate of the percentage of hospitals with pediatric inpatient hospitalizations and a lower estimate of the total number of hospitals serving pediatric patients. Additionally, the reliance on administrative data introduces the possibility of unmeasured confounding factors, and the focus on a specific timeframe may not capture the most recent trends. Readers should interpret the results with caution, recognizing these dataset constraints.

One notable limitation involves the encryption of hospital identifiers within the KID dataset. This encryption prevents linkage to external data sources, such as the AHA annual survey, which could have provided additional contextual information. Specifically, this limitation restricts our ability to differentiate between general hospitals with and without pediatric units. This constraint further emphasizes the need for caution in generalizing our findings across different hospital types and settings.

Another potential limitation of our study is the possibility of diagnostic oversight for NBSIs. While the data element PCLASS_ORPROC indicates the presence of major operating room procedures based on the Procedure Classes Refined for ICD-10-PCS, there remains a risk that physicians may overlook or misdiagnose NBSIs. This is critical given the findings from Jackson et al. (2019), which highlight deviations from WHO guidelines in diagnosing and treating neonatal sepsis [24]. Therefore, stringent adherence to diagnostic protocols is necessary to ensure that NBSI cases are accurately identified and documented. This limitation underscores the importance of developing robust diagnostic guidelines and training programs to minimize such oversight.

Furthermore, an implicit weakness of our study is that positive cultures alone do not definitively indicate disease presence. Positive cultures can sometimes be indicative of colonization rather than active infection. However, in the absence of consistent biomarkers for NBSIs, culture results remain a primary diagnostic tool. This limitation highlights the need for the development of more reliable biomarkers to improve diagnostic accuracy and patient outcomes.

### 4.3. Recommendations for Future Research

Future research should include longitudinal studies to explore temporal relationships, qualitative research to gain insights into stakeholders’ perspectives, and investigations into specific interventions aimed at reducing NBSIs. Additionally, further exploration of the impact of different regions and payment sources on hospital operations and NBSI occurrence is warranted.

## 5. Conclusions

In conclusion, this study contributes to the understanding of pediatric patient safety by identifying significant associations between patient and hospital characteristics and NBSIs. By leveraging NQI 03 as a framework, healthcare providers and policymakers can develop evidence-based strategies to enhance pediatric patient safety and mitigate the occurrence of NBSIs. Further research is needed to delve deeper into the identified associations and validate the effectiveness of targeted interventions in reducing NBSIs.

## Figures and Tables

**Table 1:** The FREQ Procedure for Neonatal Blood Stream Infections in the KID 2019 database taken from the neonatal population (AGE_NEONATE).

NBSIs	Frequency	Percent
NBSI abscent	1,833,049	78.61
NBSI present	498,829	21.39

**Table 2:** NQI 03 Neonatal Blood Stream Infection Events (PPNQ03), logistic regression (bivariate analysis) for patient characteristics, 2019.

Patient Characteristics								
Variables		Estimate	SE	Wald Chi-Square	*p*-Value	OR	Wald 95% Confidence Limits for OR	
**Gender**	Female	−0.0909	0.00231	1544.9792	0.5876	0.998	0.992	1.005
	Male ^§^	--	--	--	--	--	--	--
**Race**	Black	0.3737	0.00474	6210.2915	**<0.0001**	1.453	1.440	1.467
	Hispanic	0.3005	0.00456	4335.7171	**<0.0001**	1.351	1.339	1.363
	Asian/Pacific Islander	0.3514	0.00731	2309.3090	**<0.0001**	1.421	1.401	1.442
	Native American	−0.6356	0.0211	905.0704	**<0.0001**	0.530	0.508	0.552
	Others	0.2732	0.00656	1735.7776	**<0.0001**	1.314	1.297	1.331
	White ^§^	--	--	--	--	--	--	--
**Service line**	Maternal and Neonatal	−0.5369	0.0154	1212.1621	**<0.0001**	0.585	0.567	0.603
	Mental health/Substance use	0.2149	0.2036	1.1139	0.2912	1.240	0.832	1.848
	Injury	0.2358	0.0915	6.6453	**0.0099**	1.266	1.058	1.515
	Surgical	0.5785	0.0286	409.9134	**<0.0001**	1.783	1.686	1.886
	Medical ^§^	--	--	--	--	--	--	--
**Payment Source**	Medicare	−0.4039	0.0385	109.8998	**<0.0001**	0.668	0.619	0.720
	Medicaid	0.2659	0.00837	1009.4189	**<0.0001**	1.305	1.283	1.326
	Private insurance	0.4226	0.00837	2548.9116	**<0.0001**	1.526	1.501	1.551
	No charge	−0.5472	0.0518	111.5880	**<0.0001**	0.579	0.523	0.640
	Other	0.4556	0.0126	1304.3793	**<0.0001**	1.577	1.539	1.617
	Self-pay ^§^	--	--	--	--	--	--	--
**Operation on record**	Major operating room procedure on record	0.6831	0.0125	3003.8294	**<0.0001**	1.980	1.932	2.029

Abbreviations: CL, confidence limit; OR, odds ratio. Note: The bold *p* indicates significance (vs. the reference category) at *p* < 0.05; the symbol § indicates the reference category.

**Table 3:** NQI 03 Neonatal Blood Stream Infection Events (PPNQ03), logistic regression (bivariate analysis) for hospital characteristics.

Hospital Characteristics								
Variables		Estimate	SE	Wald Chi-Square	*p*-Value	OR	Wald 95% CL for OR	
**Hospital bed size**	Small	−0.8163	0.00485	28,347.8775	**<0.0001**	0.442	0.438	0.446
	Medium	−0.2816	0.00389	5232.2092	**<0.0001**	0.755	0.749	0.760
	Large ^§^	--	--	--	--	--	--	--
**Hospital location**	Rural	−2.6062	0.0106	60,150.4947	**<0.0001**	0.074	0.072	0.075
	Urban nonteaching	−0.7646	0.00466	26946.8518	**<0.0001**	0.466	0.461	0.470
	Urban teaching ^§^	--	--	--	--	--	--	--
**Hospital region**	Northeast	−0.0741	0.00555	178.5115	**<0.0001**	0.929	0.919	0.939
	Midwest	−0.2399	0.00509	2217.5831	**<0.0001**	0.787	0.779	0.795
	South	0.1026	0.00435	556.8232	**<0.0001**	1.108	1.099	1.118
	West ^§^	--	--	--	--	--	--	--
**Hospital ownership**	Public	−0.3219	0.00690	2177.1313	**<0.0001**	0.725	0.715	0.735
	Private, not-for-profit	0.0247	0.00499	24.5644	**<0.0001**	1.025	1.015	1.035
	Private, investor-owned ^§^	--	--	--	--	--	--	--

Abbreviations: CL, confidence limit; OR, odds ratio. Note: The bold *p* indicates significance (vs. the reference category) at *p* < 0.05; the symbol § indicates the reference category.

**Table 4:** NQI 03 Neonatal Blood Stream Infection Events (PPNQ03), logistic regression (multivariate analysis).

Variable		Estimate	SE	Wald Chi-Square Test	*p*-Value	AOR	Wald 95% CL for AOR	
**Intercept**		−0.4337	0.0194	501.5069	**<0.0001**	0.648	-	-
**Sex**	Female	0.00847	0.00351	5.8222	**0.0158**	1.009	1.002	1.015
	Male ^§^	--	--	--	--	--	--	--
**Race**	Black	0.1535	0.00517	881.5084	**<0.0001**	1.166	1.154	1.178
	Hispanic	0.0739	0.00503	215.7485	**<0.0001**	1.077	1.066	1.087
	Asian/Pacific Islander	0.0423	0.00759	31.0443	**<0.0001**	1.043	0.962	0.990
	Native American	−0.2294	0.0225	103.8504	**<0.0001**	0.795	0.761	0.831
	Others	0.0508	0.00683	55.2842	**<0.0001**	1.052	1.038	1.066
	White ^§^	--	--	--	--	--	--	--
**Service line**	Maternal and Neonatal	−0.3831	0.0161	567.0396	**<0.0001**	0.682	0.661	0.704
	Mental health/Substance use	−0.1174	0.2069	0.3217	0.5706	0.889	0.593	1.334
	Injury	−0.0435	0.0928	0.2201	0.6389	0.957	0.798	1.148
	Surgical	−0.0737	0.0329	5.0169	**0.0251**	0.929	0.871	0.991
	Medical ^§^	--	--	--	--	--	--	--
**Payment Source**	Medicare	−0.3971	0.0402	97.5495	**<0.0001**	0.672	0.621	0.727
	Medicaid	0.0759	0.00881	74.1780	**<0.0001**	1.079	1.060	1.098
	Private Insurance	0.2057	0.00884	542.0145	**<0.0001**	1.228	1.207	1.250
	No charge	−0.4010	0.0538	55.6353	**<0.0001**	0.670	0.603	0.744
	Other	0.3081	0.0133	538.8821	**<0.0001**	1.361	1.326	1.397
	Self-pay ^§^	--	--	--	--	--	--	--
**Operation on record**	Major operating room procedure on record	0.3762	0.0153	605.3762	**<0.0001**	1.457	1.414	1.501
**Hospital bed size**	Small	−0.8640	0.00502	29,669.8567	**<0.0001**	0.421	0.417	0.426
	Medium	−0.3144	0.00408	5930.7825	**<0.0001**	0.730	0.724	0.736
	Large ^§^	--	--	--	--	--	--	--
**Hospital location**	Rural	−2.5734	0.0108	57,294.8291	**<0.0001**	0.076	0.075	0.078
	Urban nonteaching	−0.7742	0.00477	26,318.7635	**<0.0001**	0.461	0.457	0.465
	Urban teaching ^§^	--	--	--	--	--	--	--
**Hospital region**	Northeast	−0.2032	0.00591	1181.1937	**<0.0001**	0.816	0.807	0.826
	Midwest	−0.1065	0.00558	364.6463	**<0.0001**	0.899	0.889	0.909
	South	0.1321	0.00474	777.7836	**<0.0001**	1.141	1.131	1.152
	West ^§^	--	--	--	--	--	--	--
**Hospital ownership**	Public	−0.3099	0.00735	1780.3272	**<0.0001**	0.733	0.723	0.744
	Private, not-for-profit	−0.0433	0.00536	65.1875	**<0.0001**	0.958	0.948	0.968
	Private, investor-owned ^§^	--	--	--	--	--	--	--

Abbreviations: CL, confidence limit; AOR, adjusted odds ratio. Note: The bold *p* indicates significance (vs. the reference category) at *p* < 0.05; the symbol § indicates the reference category.

## Data Availability

This study uses the HCUP KID dataset (2019), developed through a Federal–State–Industry partnership sponsored by the AHRQ. Data are available at https://hcup-us.ahrq.gov/databases.jsp (accessed on 19 October 2022).

## Abbreviations

AHRQ	Agency for Healthcare Research and Quality
HCUP	Healthcare Cost and Utilization Project
AE	Adverse Events
PAE	Pediatric Adverse Events
CLABSI	Central Line-associated Blood Stream Infection
KID	Kids’ Inpatient Databases
NBSI	Neonatal Blood Stream Infection
NQI 03	Neonatal Quality Indicator
PDI	Pediatric Quality Indicator
PCLASS_ORPROC	Procedure Classes Refined for ICD-10-PCS
WHO	World Health Organization
MDCs	Major Diagnostic Categories
DRG	Diagnosis-Related Group
AHA	Annual Survey of Hospitals

## Appendix A

The SAS System

The CORR Procedure

**10 Variables:**	**AGE SEX RACE I10_SERVICELINE PAY1 PCLASS_ORPROC HOSP_BEDSIZE HOSP_LOCTEACH HOSP_REGION1 H_CONTRL**									
	**Simple Statistics**									
**Variable**	**N**	**Mean**	**Std Dev**	**Sum**	**Minimum**	**Maximum**	**Label**			
**AGE**	4,571,036	5.76424	7.81784	26,348,548	0	20.00000	Age in years at admission			
**SEX**	4,569,130	1.47427	0.49934	6,736,116	1.00000	2.00000	Indicator of sex			
**RACE**	4,250,309	2.02445	1.39698	8,604,530	1.00000	6.00000	Race (uniform)			
**I10_SERVICELINE**	4,571,036	2.30483	1.74136	10,535,477	1.00000	5.00000	ICD-10-CM/PCS Hospital Service Line			
**PAY1**	4,562,501	2.63280	0.85708	12,012,167	1.00000	6.00000	Primary expected payer (uniform)			
**PCLASS_ORPROC**	4,571,036	1.88391	0.32033	8,611,429	1.00000	2.00000	Indicates operating room (major diagnostic or therapeutic) procedure on the record			
**HOSP_BEDSIZE**	4,571,036	2.28911	0.80497	10,463,605	1.00000	3.00000	Bed size of hospital			
**HOSP_LOCTEACH**	4,571,036	2.52114	0.75153	11,524,225	1.00000	3.00000	Location/teaching status of hospital			
**HOSP_REGION1**	4,571,036	2.67167	0.99289	12,212,279	1.00000	4.00000	Region of hospital			
**H_CONTRL**	4,571,036	1.99197	0.50772	9,105,379	1.00000	3.00000	Control/ownership of hospital			
	**Pearson Correlation Coefficients** **Prob > |r| under H0: Rho = 0 Number of Observations**									
	**AGE**	**SEX**	**RACE**	**I10_SERVICELINE**	**PAY1**	**PCLASS_ORPROC**	**HOSP_BEDSIZE**	**HOSP_LOCTEACH**	**HOSP_REGION1**	**H_CONTRL**
**AGE** **Age in years at admission**	1.00000 4,571,036	−0.20238 <0.0001 4,569,130	−0.04180 <0.0001 4,250,309	0.30891 <0.0001 4,571,036	−0.01622 <0.0001 4,562,501	−0.33560 <0.0001 4,571,036	−0.00704 <0.0001 4,571,036	−0.03152 <0.0001 4,571,036	−0.01026 <0.0001 4,571036	0.00198 <0.0001 4,571,036
**SEX** **Indicator of sex**	−0.20238 <0.0001 4,569,130	1.00000 4,569,130	−0.00227 <0.0001 4,248,966	0.07046 <0.0001 4,569,130	0.03537 <0.0001 4,560,603	0.06901 <0.0001 4,569,130	0.00827 <0.0001 4,569,130	0.03813 <0.0001 4,569,130	0.00893 <0.0001 4,569130	0.00039 0.4026 4,569,130
**RACE** **Race (uniform)**	−0.04180 <0.0001 4,250,309	−0.00227 <0.0001 4,248,966	1.00000 4,250,309	0.00282 <0.0001 4,250,309	−0.05614 <0.0001 4,243,736	0.00858 <0.0001 4,250,309	0.05178 <0.0001 4,250,309	0.11552 <0.0001 4250,309	0.09380 <0.0001 4,250,309	0.01458 <0.0001 4,250,309
**I10_SERVICELINE** **ICD-10-CM/PCS** **Hospital Service Line**	0.30891 <0.0001 4,571,036	0.07046 <0.0001 4,569,130	0.00282 <0.0001 4,250,309	1.00000 4,571,036	−0.00239 <0.0001 4,562,501	−0.10171 <0.0001 4,571,036	0.05416 <0.0001 4,571,036	0.12103 <0.0001 4,571,036	−0.00247 <0.0001 4,571,036	−0.03633 <0.0001 4,571,036
**PAY1** **Primary expected payer (uniform)**	−0.01622 <0.0001 4,562,501	0.03537 <0.0001 4,560,603	−0.05614 <0.0001 4,243,736	−0.00239 <0.0001 4,562,501	1.00000 4,562,501	−0.01452 <0.0001 4,562,501	−0.01467 <0.0001 4,562,501	0.01545 <0.0001 4,562,501	0.00524 <0.0001 4,562,501	0.00819 <0.0001 4,562,501
**PCLASS_ORPROC** **Indicates operating room (major diagnostic or therapeutic) procedure on the record**	−0.33560 <0.0001 4,571,036	0.06901 <0.0001 4,569,130	0.00858 <0.0001 4,250,309	−0.10171 <0.0001 4,571,036	−0.01452 <0.0001 4,562,501	1.00000 4,571,036	−0.00590 <0.0001 4,571,036	−0.02304 <0.0001 4,571,036	−0.01690 <0.0001 4,571,036	−0.01271 <0.0001 4,571,036
**HOSP_BEDSIZE** **Bedsize of hospital**	−0.00704 <0.0001 4,571,036	0.00827 <0.0001 4,569,130	0.05178 <0.0001 4,250,309	0.05416 <0.0001 4,571,036	−0.01467 <0.0001 4,562,501	−0.00590 <0.0001 4,571,036	1.00000 4,571,036	0.05958 <0.0001 4,571,036	0.06144 <0.0001 4,571,036	−0.07654 <0.0001 4,571,036
**HOSP_LOCTEACH** **Location/teaching status of hospital**	−0.03152 <0.0001 4571,036	0.03813 <0.0001 4,569,130	0.11552 <0.0001 4,250,309	0.12103 <0.0001 4,571,036	0.01545 <0.0001 4,562,501	−0.02304 <0.0001 4,571,036	0.05958 <0.0001 4,571,036	1.00000 4,571,036	−0.04933 <0.0001 4,571,036	0.05362 <0.0001 4,571,036
**HOSP_REGION1** **Region of hospital**	−0.01026 <0.0001 4,571,036	0.00893 <0.0001 4,569,130	0.09380 <0.0001 4,250,309	−0.00247 <0.0001 4,571,036	0.00524 <0.0001 4,562,501	−0.01690 <0.0001 4,571,036	0.06144 <0.0001 4,571,036	−0.04933 <0.0001 4,571,036	1.00000 4,571,036	0.03591 <0.0001 4,571,036
**H_CONTRL** **Control/ownership of hospital**	0.00198 <0.0001 4,571,036	0.00039 0.4026 4,569,130	0.01458 <0.0001 4,250,309	−0.03633 <0.0001 4,571,036	0.00819 <0.0001 4,562,501	−0.01271 <0.0001 4,571,036	−0.07654 <0.0001 4,571,036	0.05362 <0.0001 4,571,036	0.03591 <0.0001 4,571,036	1.00000 4,571,036

## Appendix B

### Appendix B.1. Further Variable Description for Selected Variables

- The hospital’s bed size category (H_BEDSZ) is nested within location and teaching status (H_LOCTCH) [18].

**Location and Teaching Status**	**Bed Size**		
	**Small**	**Medium**	**Large**
Rural	1–49	50–99	100+
Urban, nonteaching	1–99	100–199	200+
Urban, teaching	1–299	300–499	500+

B. The data element PCLASS_ORPROC indicates whether a major operating room procedure was reported on the discharge record [18].

The presence of operating room procedures reported on a discharge record was determined using the Procedure Classes Refined for ICD-10-PCS. Each ICD-10-PCS procedure code on the record was classified into one of four procedure classes:

- Minor Diagnostic: Non-operating-room procedures that are diagnostic (e.g., BW2800Z CT scan of head using high osmolar contrast);
- Minor Therapeutic—Non-operating-room procedures that are therapeutic (e.g., 079030Z drainage of head lymph, percutaneous approach);
- Major Diagnostic—All procedures considered valid operating room procedures by the Diagnosis-Related Group (DRG) grouper and that are performed for diagnostic reasons (e.g., 00B00ZX excision of brain, open approach, diagnostic);
- Major Therapeutic—All procedures considered valid operating room procedures by the Diagnosis-Related Group (DRG) grouper and that are performed for therapeutic reasons (e.g., 021008W bypass coronary artery, one artery from aorta with zooplastic tissue, open approach).

If at least one major diagnostic or major therapeutic procedure was included on the record, then PCLASS_ORPROC was set to 1. More information on the Procedure Classes (https://hcup-us.ahrq.gov/toolssoftware/procedureicd10/procedure_icd10.jsp) is available under Tools & Software on the HCUP-US website (accessed on 29 October 2022).

Prior to the data year 2020, the major operating room procedure indicators were stored in the data element I10_ORPROC.

**Variable**	**Description**	**Value**	**Value Description**
PCLASS_ORPROC	Major operating room ICD-10-PCS procedure indicator	0	No major operating room procedure reported on discharge record
		1	Major operating room procedure reported on discharge record

C. All discharges were categorized into five hospitalization types (i.e., service lines) in the following hierarchical order: Maternal/Neonatal, Mental health/Substance abuse, Injury, Surgical, and Medical. The criteria for identifying the hospitalization types varies across data years.

### Appendix B.2. Beginning in Data Year 2019

Beginning in the data year 2019, the five hospitalization types were defined as follows:

Maternal and neonatal discharges were defined using the following MDCs:

- MDC 14 Pregnancy, Childbirth, and Puerperium
- MDC 15 Newborn and Other Neonates (Perinatal Period)

Mental health/Substance use discharges were defined using the following MDCs:

- MDC 19 Mental Diseases and Disorders
- MDC 20 Alcohol/Drug Use or Induced Mental Disorders

Injury discharges were identified using the following screen:

- Clinical Classification Software-Refined categories for the principal ICD-10-CM diagnosis:
  ○ INJ001–INJ027
  ○ INJ032

Surgical discharges were identified by a surgical DRG, the same definition used prior to the data year 2019. The DRG grouper first assigns the discharge to an MDC based on the principal diagnosis. For each MDC, there is a list of procedure codes that qualify as operating room procedures. If the discharge involved an operating room procedure, wat is assigned to one of the surgical DRGs within the MDC category.

All other discharges that were not assigned to a surgical DRG were identified as a Medical discharge. If the DRG indicated the information on the record was ungroupable (i.e., not identifiable as medical or surgical), then the discharge was assumed to be medical. This rarely occurred (less than 0.1 percent of total discharges).

It should be noted that discharges with a principal diagnosis of injury or poisoning by intentional self-harm are classified under the Medical service line and not the Mental health/Substance use or Injury service lines [18].

### Appendix B.3. Dependent Variable Definition

**Table A1:** AHRQ Hospital-level PDI Descriptions and Numerators (AHRQ, 2021).

PDI Number	PDI Name	PDI Description	PDI Numerator
**NQI 03**	**Neonatal Blood Stream Infection Rate (https://qualityindicators.ahrq.gov/Downloads/Modules/PDI/V2019/TechSpecs/NQI_03_Neonatal_Blood_Stream_Infection_Rate.pdf accessed on 29 October 2022)**	Discharges with healthcare-associated blood stream infection per 1000 discharges for newborns and outborns with birth weight of 500 g or more but less than 1500 g; with gestational age between 24 and 30 weeks; or with birth weight of 1500 g or more, and death, an operating room procedure, mechanical ventilation, or transferring from another hospital within two days of birth. Excludes discharges with a length of stay less than 3 days and discharges with a principal diagnosis of sepsis, bacteremia, or newborn bacteremia.	Discharges among cases meeting the inclusion and exclusion rules for the denominator, with either: • any secondary ICD-10-CM diagnosis codes for newborn sepsis (BSI5DX) or • any secondary ICD-10-CM diagnosis codes for newborn septicemia or bacteremia codes requiring a separate organism code (BSI2DX) and any secondary ICD-10-CM diagnosis codes for staphylococcal or Gram-negative bacterial infection (BSI3DX)

## Appendix C. Descriptive Number of Observations of Each Tested Independent Variable in the HCUP KID Database

**Hospital Characteristics**				
**Variable Name**	**Level**	**Number of Observations**	**Percent %**	**Missing %**
**Hospital bed size**	Small	470,770	15.2	0.0
	Medium	742,057	24.0	
	Large	1,876,456	60.7	
**Hospital location**	Rural	189,298	6.1	0.0
	Urban nonteaching	356,963	11.6	
	Urban teaching	2,543,022	82.3	
**Hospital region**	Northeast	529,073	17.1	0.0
	Midwest	696,645	22.6	
	South	1,183,705	38.3	
	West	679,860	22.0	
**Hospital ownership**	Public	365,784	11.8	0.0
	Private, not-for-profit	2,382,758	77.1	
	Private, investor-owned	340,741	11.0	
***Patient Characteristics***				
**Gender**	Female	1,587,394	51.4	0.0
	Male	1,500,745	48.6	
**Race**	White	1,407,652	45.6	7.2
	Black	513,619	16.6	
	Hispanic	607,329	19.7	
	Asian/Pacific Islander	123,698	4.0	
	Native American	26,306	.9	
	Other	188,042	6.1	
**Service line**	Maternal and Neonatal	1,716,825	55.6	0.0
	Mental health/Substance use	209,939	6.8	
	Injury	97,434	3.2	
	Surgical	219,576	7.1	
	Medical	845,509	27.4	
**Payment source**	Medicare	10,554	0.3	0.1
	Medicaid	1,567,452	50.7	
	Private Insurance	1,270,547	41.1	
	Self-pay	131,918	4.3	
	No charge	3843	0.1	
	Other	100,660	3.3	
**Operation on record**	No operation on record	2,718,390	88.0	0.0
	Major operation on record	370,893	12.0	
